# The Effects of Supplemented Conjugated Linoleic Acid on Lipid Metabolism in Cattle

**DOI:** 10.3390/ani16040550

**Published:** 2026-02-10

**Authors:** Cheng Xiao, Elke Albrecht, Harald M. Hammon, Steffen Maak

**Affiliations:** 1 Research Institute for Farm Animal Biology (FBN), 18196 Dummerstorf, Germany; xiao@fbn-dummerstorf.de (C.X.); hammon@fbn-dummerstorf.de (H.M.H.); maak@fbn-dummerstorf.de (S.M.); 2 Institute of Animal and Veterinary Sciences, Jilin Academy of Agricultural Sciences, Gongzhuling 136100, China; 3 Faculty of Agricultural and Environmental Sciences, University of Rostock, 18057 Rostock, Germany

**Keywords:** body composition, cattle, cell, conjugated linoleic acid, energy balance, lactation, milk fat depression, transition

## Abstract

Conjugated linoleic acid (CLA) is produced by bacterial biohydrogenation in the rumen of cattle, fulfills various biological functions, and is known for anti-obesity, anti-inflammation, anti-cancer, and other beneficial effects. It has numerous isomers, of which cis-9,trans-11 CLA accounts for 80% of total CLA, followed by trans-10,cis-12 CLA (t10,c12 CLA), with distinct molecular structures, oxidation efficiencies, activities, and functions. Different effects were observed, when isomers were individually supplemented in livestock nutrition. Currently, CLA is supplemented into the diets of dairy cows to improve the energy balance, and avoid negative effects of energy loss during the transition period. Furthermore, t10,c12 CLA was shown to reduce subcutaneous fat and to improve intramuscular fat (IMF) content in the carcasses of ruminants and pigs. Increasing the IMF content without increasing other fat depots and without compromising feed efficiency is an important goal in beef production. However, inconsistent and conflicting results were reported partly based on different study designs. This review aims to summarize studies on CLA supplementation in cattle, focusing on t10,c12 CLA and the effects of the dose, time, and method of supplementation on energy balance, milk yield and body composition, as well as on individual cells in vitro. This may improve our understanding of energy-saving and repartitioning effects of CLA in cattle.

## Simple Summary

Milk and meat products of cattle are the main source of conjugated linoleic acid (CLA) for human nutrition. Increasing the amount of CLA in milk and meat products may be beneficial for consumers but is associated with changes in lipid metabolism of supplemented animals, which are not completely understood. Currently, CLA is supplemented into diets of high yielding dairy cows to reduce their energy deficit, when they are not able to meet the high energy demand of milk production by increased feed intake. Milk fat depression by CLA can help to avoid negative effects of energy loss and mobilization of body reserves for lactation. Furthermore, CLA was shown to reduce waste fat and to improve marbling fat in ruminants and pigs. Increasing the muscle fat content and improving meat quality without increasing other fat depots and without compromising feed efficiency are important goals in beef production. Additionally, increased CLA in meat makes it a healthier product for human nutrition. However, inconsistent results and higher costs limit the practical application of CLA supplementation. This review summarizes the current knowledge about the effects of supplemented CLA in dairy cows and beef cattle, as well as on individual cells in cell culture models.

## 1. Introduction

The contents of fat and protein in milk and in the carcass are important economic factors in dairy and beef production. In early lactation, cows often cannot meet the energy requirement for milk synthesis by increased feed intake. Thus, body fat is mobilized [1], which can cause health problems, diseases, and even death in cows [2]. To alleviate the energy problems of cows during the transition period, increasing the energy density of diets is feasible but limited by the feed intake capacity of the lactating cows, effects on dry matter intake (DMI), digestibility, milk protein content, etc. [3]. The change from pasture-based feeding systems to corn silage-based diets or fat supplementation to improve the energy intake of high-yielding dairy cows could only partly solve this problem. However, it was observed that particular diets, which increase the conjugated linoleic acid (CLA) content in mammary gland and other tissues, caused a low-fat milk syndrome, later referred to as milk fat depression (MFD). This reduction in the milk fat content can efficiently improve the energy balance in early lactating cows, as milk fat synthesis requires up to 50% of the energy demand for total milk production [4]. To achieve this, various feed additives were tested to increase the endogenous CLA production or rumen-protected CLA was directly added to the diet [5]. Such a feeding strategy was shown to improve the energy balance due to MFD [6,7].

In beef cattle, high-energy feeding during the finishing period is applied to increase intramuscular fat deposition in order to improve meat quality. Excessive nutritional energy is stored as fat in white adipose tissue—an indispensable organ in livestock and humans—which fulfills various physiological functions, avoids lipotoxicity, provides energy by lipolysis during exercise and fasting, and secretes hormones to regulate biological processes [8]. White adipose tissue is distributed over the whole body and forms fat depots, including subcutaneous, intramuscular, intermuscular, retroperitoneal, mesenteric and omental fat, with different composition, gene expression, and biological functions [9]. In livestock, subcutaneous fat is beneficial to maintain the water content and value of carcasses, however excessive fat accumulation in subcutaneous fat leads to increased waste and economical losses [10,11]. Some studies found that CLA supplementation reduced subcutaneous fat deposition and increased intramuscular fat content, and thus, had positive effects on meat quality in cattle and pigs [12,13].

Conjugated linoleic acid is a family of fatty acids containing 18 carbon atoms and conjugated double bonds, and theoretically includes 56 isomers [14]. The most abundant isomer is cis-9/trans-11 CLA (c9,t11 CLA), which acts as an anti-carcinogenic, followed by trans-10/cis-12 CLA (t10,c12 CLA), which is thought to be involved in nutrient partitioning between fat and muscle [15,16]. Although the two CLA isomers are both derived from biohydrogenation of linoleic acid (LA) and α-linolenic acid (ALA) in the rumen, they have distinct molecular structures, which enable distinct biological functions, oxidation efficiencies, and bioactivities [17,18]. The regulatory mechanisms of CLA isomers on the lipid metabolism is not fully understood and there are inconsistent and partly controversial results, probably due to the distinct percentage of supplemented CLA isomers, the animal species, or the physiological stage of the animals. Consequently, this review summarizes the results of relevant studies on CLA supplementation effects in cattle aiming at elucidation of the metabolism and function of CLA in blood, milk, mammary gland, and adipose tissue, thereby focusing on the metabolically more active t10,c12 isomer. Moreover, effects of dose, time, and method of supplementation will be discussed as well as relevant genes and regulatory pathways. Finally, studies which investigated the direct effects of CLA isomers on individual cells using cell culture models were considered. This may give a new perspective and better understanding of metabolic changes by CLA supplementation in cattle and their cellular basis.

## 2. Conjugated Linoleic Acid Formation and Metabolism

Essential fatty acids, such as linoleic acid (LA) and α-linolenic acid (ALA), cannot be synthesized by endogenous metabolism and must be absorbed with food [19]. Vegetables, crops, and grass are rich in LA or ALA that are finally biohydrogenated by bacteria in the rumen into stearic acid (C18:0) [20]. However, during this process of biohydrogenation, a part of LA can be metabolized into c9,t11 CLA and is transported from the rumen to other tissues, whereas a part of ALA can be converted into vaccenic acid (C18:1 trans-11). Vaccenic acid in adipose tissue and mammary tissue can also be a source of endogenously synthesized c9,t11 CLA by Δ9 desaturase as shown in lactating dairy cows [21]. A study in rats that were supplemented with vaccenic acid confirmed that Δ9 desaturase also metabolized the exogenous vaccenic acid into c9,t11 CLA [22]. Thus, this metabolic process may be consistent in different animal species. Since the Δ12 desaturase enzyme does not exist in mammals, the vaccenic acid cannot be metabolized into t10,c12 CLA and this isomer is directly transferred from the rumen to peripheral tissues [23]. C9,t11 CLA, and t10,c12 CLA are the most abundant isomers of CLA, of which the c9,t11 isomer accounts for 80% of total CLA, because of the high expression of Δ9 desaturase in adipose and mammary tissues [24]. T10,c12 CLA is less abundant but has a higher bio-activity than c9,t11 CLA [23].

The distinct molecular structures of c9,t11 CLA and t10,c12 CLA facilitate different cellular accumulation and incorporation of these isoforms. The cis configuration leads to linear fatty acids with a bend degree; this shape is detrimental for accumulation in triglycerides, but maintains the fluidity and curvature of the membranes [25]. Thus, the cis configuration is preferentially incorporated into phospholipids of organelle membranes and polyunsaturated fatty acids (PUFA); LA, and ALA are enriched in membrane phospholipids [26]. The structure of c9,t11 CLA is similar to that of LA (C18:2 cis-9,cis-12) and is also preferentially incorporated into phospholipids of organelle membranes [27]. The trans bond configuration of t10,c12 CLA has a slight curvature similar to the structure of monounsaturated fatty acids (MUFA) and supports the preferential incorporation into triglycerides [25]. A study in beef cattle showed that t10,c12 CLA is preferentially stored in subcutaneous fat [28]. Consequently, manipulation of t10/c12 CLA concentration in adipose and other tissues can more easily be achieved, and its effects can more easily be studied, than that of c9,t11 CLA; however, accumulation in tissues may cause long term effects which need to be explored.

## 3. Effects of CLA Supplementation on Cows During the Transition Period

### 3.1. Transfer Efficiency of Supplemented CLA

High-yielding dairy cows, such as Holstein cows, are raised all over the world. When dairy cows experience negative energy balance during the transition period and cannot consume sufficient energy to meet the energy demand of high milk production, they mobilize body reserves, which may result in adverse health effects [29]. Studies demonstrated that supplementation with t10,c12 CLA can significantly reduce the content of milk fat and repartition energy in the cow’s body, with positive health effects [30]. However, as considered in the following, inconsistent results were achieved due to variation in type, period, duration, and dose of CLA application, analyzed tissues and further variables, such as breed, parity and diet composition.

*Type of supplementation*: The CLA supplementation method affects the transfer ratio and efficacy of t10,c12 CLA in the tissues. Several studies showed that dietary CLA was digested by microbes in the rumen, even though it was provided as rumen-protected CLA. Only a part of CLA reached the small intestine and was absorbed by other tissues. Sigl and colleagues [31] showed that 38.5% of rumen-protected dietary CLA was digested by microbes in the rumen. Moreover, more than 80% of CLA calcium salts were digested by microbes in the rumen in the study of Moallem et al. [32]. De Veth et al. [33,34] and Perfield et al. [35] showed that only 2.6%, 3.8% and 7.9% of the supplemented CLA, respectively, were transferred into milk fat. In contrast, the experimental method of abomasal infusion avoids fermentation by microbes in the rumen and more efficiently transfers CLA into the tissues. Chouinard and colleagues showed that the transfer ratio of c9,t11 CLA and t10,c12 CLA into milk fat by this method was 22.5% and 10.2%, respectively [17]. Consequently, abomasal infusion of t10,c12 CLA results in MFD within 24 h [36]. When comparing the transfer efficiency of CLA into milk fat, it is obvious that the infusion method results in a higher transfer ratio of t10,c12 CLA and is more effective than dietary supplementation in cows during the transition period. However, this can only be an experimental approach, while in practice, dietary application is merely feasible.

*Dosage*: CLA affects milk and blood composition, and the lipid metabolism of cows in a dose-dependent manner. CLA supplementation studies indicate that the transfer ratio of CLA into milk is dose-dependent. In late lactating cows, Chouinard et al. [17] reported a dose-dependent change in milk and desaturase index, when three different doses of CLA (31.3, 57.7, and 90.0 g/d mixture of 4 isomers) were supplemented by infusion. Moore et al. demonstrated the decrease in the milk fat content with an increase in the supplemented dose of dietary rumen-protected CLA (62, 125, and 187 g/d) [5]. The study of Haubold et al. [37] in lactating dairy cows confirmed the dose-dependent extent of milk fat depression, when cows were supplemented by abomasal infusion with three different doses of CLA (16, 32, and 64 g/d of Lutalin, BASF).

*Time*: In the lactating cows, transfer efficiency and function of the CLA supplementation depends on the stage of lactation. For example, the study of Galamb et al. [38] demonstrated that CLA supplementation during the transition period was more efficient in MFD and decreased body fat mass mobilization was stronger than supplementation starting after calving. Furthermore, the duration of supplementation influences the efficacy. Studies using supplementation of t10,c12 CLA in the 3T3-L1 cell line and mice for different durations showed that the number of differentially expressed genes (DEGs) increased in cells and white adipose tissue in a time-dependent manner [39,40,41,42]. Further exploration is needed to investigate if this effect is also relevant in the individual cells of cow tissues.

*Storage and distribution*: T10,c12 CLA can be detected in mammary gland and retroperitoneal fat tissue after 42 days of supplementation and in other fat tissues also after 105 days, but not in muscle tissue [43]. Supplemented t10,c12 CLA, together with the majority of fatty acids from body fat mass mobilization, is transferred into the mammary gland or tissues according to the energy and metabolic needs. Retroperitoneal fat tissue appears to be more sensitive than other adipose depots to body mass mobilization during early lactation in cows, and its weight decreased to 47.7% at 42 days in milk in control cows compared to cows slaughtered one day after calving in the study of von Soosten et al. [44]. CLA supplemented cows showed a reduced retroperitoneal fat tissue loss by 34% compared to controls in this study. Without supplementation, t10,c12 CLA could only be detected at day 105 of lactation in the retroperitoneal fat tissue of the control group [43]. Thus, a significant contribution of t10,c12 CLA derived by tissue mobilization cannot be expected. However, supplementation of CLA was associated with reduced loss of adipose tissue while inhibiting body fat mass mobilization [43,44]. Concordantly, the study of Dirandeh et al. [45] indicated that dietary CLA supplementation affected lipid metabolism in subcutaneous fat through upregulating and downregulating of genes involved in lipogenesis and lipolysis, respectively.

### 3.2. Effects of CLA Supplementation on Milk Composition

Studies indicated that the supplementation of lactating dairy cows with t10,c12 CLA, in contrast to supplementation with c9,t11 CLA, leads to MFD [46]. Mainly consistent results of t10,c12 CLA supplementation effects on milk fat content and yield were reported and are summarized in Table 1. Mammary epithelial cells (MECs) are the center of milk fat synthesis and synthesize de novo fatty acids and triglycerides to form milk fat globules (MFGs) [47,48,49]. These MFGs are the main structure of milk fat, and their size and number are affected by breed, genetics, and diet [50,51,52]. Xing et al. [50] and Zhang et al. [53] showed that MFD by t10,c12 CLA leads to decreased number, reduced diameter, and enhanced percentage of small-sized MFGs. The fatty acids of MFGs are mainly derived from de novo fatty acid synthesis, which is decreased by t10,c12 CLA (Table 1). The study of Haubold et al. [37] indicated that CLA led to MFD by reducing the content of fatty acids with less than 16 carbons in milk. This indicates reduced de novo fat synthesis, because short-chain fatty acids (4 to 8 carbons) and medium-chain fatty acids (10 to 14 carbons) arise almost exclusively from de novo synthesis [4]. Zhang et al. [54] treated goat mammary epithelial cells with t10,c12 CLA and found that de novo fatty acid synthesis is decreased by inhibition of gene expression of *FASN*, *SREBP1*, and *ACACA*. In biopsies of mammary gland tissue, Baumgard et al. [55] observed a reversible and coordinated decrease in gene expression for key enzymes involved in milk fat generation after treatment with t10,c12 CLA. Thus, milk fat yield and composition are changed accordingly.

Milk fat accounts for 50% of total energy necessary for milk production, thus MFD by t10,c12 CLA decreases milk energy output and body fat mass mobilization, and saves energy in cows during the transition period [56]. Vogel et al. [56] concluded that milk citrate content increased in the CLA supplemented cows indicating t10,c12 CLA-mediated energy-saving, because citrate is a substrate for de novo fatty acid synthesis and energy production [57]. According to the studies summarized in Table 1, the saved energy mainly flows into three different directions, energy balance, body composition, or milk yield. Most studies showed that t10,c12 CLA supplementation decreased milk energy output; thus, the energy balance of the body is improved [56,58]. This is the common direction of saved energy and the reason why the t10,c12 CLA supplementation may be recommended as a feeding strategy for cows during the transition period [59]. However, the response to CLA supplementation varies among cows and the anticipated effect is not always observed. The secondary direction of saved energy is body mass development or change in body composition. The study of Xiao et al. [60] indicated increased body mass, intramuscular fat content, and lean meat mass by CLA supplementation. The third direction of saved energy is milk yield. Thus, total milk yield and sometimes the content or yield of milk protein is increased [61]. The different flow directions of saved energy may lead to controversial results in DMI, energy balance, milk yield, milk protein content and yield upon CLA supplementation. Remarkably, the energy balance in medium or late lactating cows is positive, and CLA supplementation decreases the milk fat content without changing the energy balance (Table 1). Therefore, CLA supplementation has beneficial effects in cows mainly during the transition period [62], even though effects may be obvious after a few days [56].

Some studies showed that CLA supplementation might affect the palatability of the diet and lead to a change in the DMI of cows. However, the same palatability of diet with CLA still led to an increase or decrease in DMI in the study of Moore et al. [5]. Type of dietary supplementation, as well as abomasal infusion of CLA, can change the DMI of cows [32,56], indicating that the DMI is orchestrated according to the energy level in order to maintain the energy balance.

**Table 1:** The effects of t10,c12 CLA supplementation, either dietary or via abomasal infusion, on milk composition, dry matter intake (DMI), body weight (BW), and energy balance (EB).

Cows	Supplementation, g/d	Method	Period	Milk Fat Content	Milk Fat Yield	Milk Yield	Milk Protein Content	Milk Protein Yield	DMI	EB	BW	ECM	De Novo FA	Reference
m-Holstein	10.2	infusion	−63 d to 63 d	↓	↓	↔	↓	↔	↓	↑	↔	↓	↓	[56]
m-Holstein	4.9/9.9/14.8	dietary	−10 d to 21 d	↓	↓	↔	↔	↔	↔	↔	↔	/	↓	[5]
p-Brown Swiss	3.75/10	dietary	−14 d to 28 d	↔	↔	↔	↔	↔	/	/	/	/	/	[31]
m-Holstein	7.5	dietary	−20 d to 21 d	↓	↓	↔	↔	↔	↔	/	↑	/	/	[45]
m-Holstein	6.8	dietary	−21 d to 28 d	↔	↔	↔	↔	↔	↑	↔	↑	↔	/	[62]
m-Holstein	12	dietary	−21 d to 60 d	↓	↓	↑	↔	↑	↔	↔	↔	↔	/	[61]
m-Holstein	6.8	dietary	−21 d to 91 d	↓	↔	↑	↔	↔	↑	/	/	↔	/	[38]
pm-Holstein	3.8	dietary	−21 d to 98 d	↓	↓	↑	↓	↔	↔	↑	/	↓	/	[63]
p-Holstein	6	dietary	1 d to 105 d	↓	↓	↔	↓	↔	↔	↔	↔	↔	/	[44]
m-Holstein	4.7	dietary	21 d to 100 d	↓	↓	↑	↓	↔	↓	↔	↓	↓	↓	[32]
m-Holstein	140	dietary	136 d for 7 d	↓	↓	↔	↔	↔	↔	/	/	/	↓	[53]
m-Holstein	88	dietary	145 d for 6 d	↓	↓	↔	↔	↔	↔	/	/	/	/	[50]
m-Holstein	4.6/9.2/18.4	infusion	126 d for 6 w	↓	↓	↔	↓	↔	↔	↔	↔	↓	↓	[37]
m-Holstein	10.8/19.9/31	infusion	258 d for 10 d	↓	↓	↔	↔	↔	↔	/	/	/	↓	[17]
m-Holstein	13.6	infusion	286 d for 5 d	↓	↓	↔	↑	↓	↔	/	/	↓	↓	[55]

DMI: dry matter intake; EB: energy balance; BW: body weight; ECM: energy corrected milk; FA: fatty acids; m: multiparous; p: primiparous; /: no data; ↓ decrease; ↑ increase; ↔ no change.

### 3.3. Effects of CLA Supplementation on Circulating Compounds and Liver Triglycerides

The high demand for energy, glucose, and protein for milk production and the often insufficient feed intake in the first weeks after calving are reflected by a drop in plasma glucose and insulin concentrations and an increase in plasma concentrations of non-esterified fatty acids (NEFA) and β-hydroxybutyric acid (BHBA) as well as fat in the liver [64,65,66]. The increase in NEFA concentrations is an expression of body fat mobilization, whereas the increase in BHBA indicates the incomplete oxidation of fatty acids and the formation of ketone bodies in the liver [1]. At this period, high amounts of NEFA are taken up by the liver, re-esterified to triglycerides and stored [1]. Therefore, excess body fat mass mobilization leads to fatty liver in cows during the transition period [67]. Supplementation with t10,c12 CLA can contribute to avoiding the respective metabolic imbalances. Table 2 gives an overview of the changes in blood and the liver during early lactation and the effects of CLA supplementation. Partially conflicting results were observed, depending on the amount and type of supplementation, time frame of treatment and other unknown factors. It was shown that abomasal infusion of CLA effectively increased the content of t10,c12 CLA in plasma [68]. Moreover, t10,c12 CLA reduced the content and yield of milk fat and body fat mass mobilization, so the content of NEFA in plasma in the CLA supplemented group was lower than in the control group in the study of Vogel et al. [56]. The lower body fat mobilization after CLA administration after calving also leads to less fat storage in the liver as indicated by lower hepatic triglyceride content in cows treated with CLA [56]. Furthermore, CLA supplementation relieves the glucose metabolism of dairy cows; the glucose concentration in blood plasma is higher, similar to the glycogen content in the liver, in cows that receive CLA during the transition phase [69]. Cows treated with CLA show a reduced endogenous glucose production and glucose oxidation. CLA treatment therefore has a glucose-saving effect on the metabolism of dairy cows during early lactation [69,70]. The relief of energy metabolism after CLA administration is also evident from the stimulation of plasma insulin, which activates the somatotropic axis [71]. Thus, the content of glucagon and cortisol in plasma and the ratio of glucagon/insulin and glucose/insulin remained low in the CLA supplemented cows. It was shown in the study of Morais [72] that decreased cortisol content in the blood may improve insulin sensitivity. Higher plasma concentrations of insulin, IGF-I and the ratio of the binding proteins IGFBP-3/IGFBP-2 in blood plasma, as well as corresponding changes in gene expression in the liver, support this statement. In summary, t10,c12 CLA-mediated MFD and less body fat mass mobilization can lead to lower concentrations of NEFA, glucagon, and cortisol and higher concentrations of glucose, insulin, and IGF-I in plasma, and reduces the triglycerides in the liver during the transition period. However, the high variability among studies and individual cows may lead to inconsistent results and long-term consequences of observed changes should be investigated in the future. It should be noted that the supplemented fatty acids are transferred to the offspring via the placenta, as shown in the study of Uken et al. [73] and may result in comparable metabolic changes that affect the development of the fetus and neonate. This will be considered later in more detail.

### 3.4. Changes in Mammary Epithelial Cells by CLA Supplementation

Mammary epithelial cells (MECs) synthesize de novo fatty acids and take up long-chain fatty acids (LCFAs) from blood for milk fat production [49]. The transfer of CLA into the mammary epithelial cells is a prerequisite for effects on MFD. In a study by Zhang et al. [54], goat MECs were treated for 12 h with 100 μM or 200 μM t10,c12 CLA to detect differentially expressed genes (DEGs) in comparison to non-supplemented control cells. They detected 49 DEGs in cells supplemented with 100 μM CLA and 854 DEGs in cells supplemented with 200 μM CLA compared to control cells. Clear dose-dependent effects on genes related to fatty acid metabolism could be confirmed with RT-qPCR [54].

Studies showed that t10,c12 CLA reached MECs and regulated transcription factors, genes, and proteins that inhibit de novo fatty acid synthesis, such as ACLY, ACC, and FAS [54,77,78]. On the contrary, the percentage of LCFAs increased in milk fat. LCFAs are packed into very low-density lipoprotein (VLDL) in the liver, or chylomicrons in intestinal cells, and are delivered into other tissues by bonding with apolipoproteins [79]. Veshkini et al. [80] showed that the apolipoproteins in blood, such as APOA1, APOA4, and APOC3, increased in the CLA-supplemented cows. The APOC3, APOA1, and APOA4 are correlated with VLDL, HDL (high density lipoprotein), and chylomicrons, respectively [80,81,82]. CD36 protein in MECs is responsible for transferring LCFAs into organelles, and accordingly, t10,c12 CLA increased *CD36* expression in MECs [77]. Transcription factors in MECs determine lipogenesis, lipolysis, and fatty acid synthesis. PPARγ and SREBP1 are crucial transcription factors for regulating adipogenesis and lipogenesis [83]. Studies showed that t10,c12 CLA inhibits *PPARG* and *SREBP1* and their downstream genes, including *ACACA*, *FASN*, *SCD*, *LPL*, and *FABP4* in MECs [77,84,85]. T10,c12 CLA also stimulated lipolysis by regulating CPT1, HSL, and PLIN1 proteins. AMPK, mTOR, and THRSP signaling pathways are all affected by t10,c12 CLA and participate in MFD [86]. A summary and schematic representation of the involved genes, proteins, lipids and pathways are presented in [59].

### 3.5. Changes in Adipose Tissue by CLA Supplementation

The t10,c12 CLA is preferentially stored in adipose tissues, and the effects of CLA on adipose tissues are key for exploring the regulatory mechanisms involved in inhibiting fatty acid synthesis and fat accumulation by t10,c12 CLA. Akter et al. [87] reported that the body fat mass mobilization in cows during the transition period mainly occurred in retroperitoneal, omental, mesenteric, and subcutaneous fat tissues, of which the retroperitoneal fat responded early with mobilization. The adipocyte size of the retroperitoneal fat was bigger than that of other fat tissues, and its weight and adipocyte size decreased first after calving [87]. The mobilization of retroperitoneal fat was positively associated with the content of NEFA in plasma in the early lactating cows [87]. With the increase in body mass mobilization, the weight and adipocyte size of other fat depots decreased [43,44,87].

The amount of supplemented t10,c12 CLA that reaches a tissue may vary due to individual conditions, such as genetics and actual energy deficit. The effects on adipose tissue mobilization, resulting in weight loss and decreased adipocyte size may vary accordingly. Thus, in the study of Xiao et al. [60], no changes in the size of subcutaneous adipocytes were observed compared to control cows, when cows were supplemented with CLA from 63 d antepartum to 63 d postpartum. However, changes occurred in gene expression related to adipocyte differentiation and lipogenesis, which demonstrated that CLA still affected lipid metabolism in the subcutaneous fat tissue. CLA was shown to inhibit lipogenesis and adipocyte differentiation in cells and adipose tissues by reducing *PPARG*, *SREBP1*, *ACACA*, *FASN*, *SCD*, *LPL*, and others [49,54,84,88]. In contrast, Xiao et al. [60] measured increased *LPL* and *FASN* expression in the subcutaneous adipose tissue of CLA supplemented cows. LPL protein catalyzes and facilitates the uptake of fatty acids into tissues [89], whereas ACACA and FASN are limiting enzymes for de novo fatty acid synthesis [90,91]. However, the adipocyte size of subcutaneous fat did not change. Xiao et al. [60] also reported improved energy balance and increased intramuscular fat (IMF) content in CLA supplemented cows, while the expression of *PPARG*, *CEBPA*, and *SREBP1*, indicating development of new adipocytes, did not change in the longissimus muscle. However, the expression of *ACACA* and *FASN* increased and improved IMF deposition. Daddam et al. [58] reported that both lipogenesis and lipolysis were stimulated by CLA supplementation. Even though, CLA inhibited fat synthesis, it appeared that lipogenesis was also stimulated in cows since more fat was stored due to the improved energy balance. The stage of lactation may be responsible for the differing results, if cows are still in negative energy balance or start to recover and repartitioning surplus energy to rebuild adipose tissues.

### 3.6. Changes in Muscle Tissue by CLA Supplementation

In addition to the negative energy balance, cows may develop a negative protein balance during the transition period [92]. Thus, muscle mass is mobilized contributing free amino acids (AA) to the plasma pool for the onset of lactation as a mechanism of normal metabolic adaptation [93]. Free AA can be used for several biological processes including milk protein synthesis, direct oxidation, or gluconeogenesis, and others [93]. Most studies used ultrasonography or measurement of serum concentration of creatinine and plasma concentration of 3-methylhistidine to estimate muscle tissue mobilization [94]. The main structural component of muscle tissue are muscle fibers, whose number is fixed at birth, but muscle fiber hypertrophy and conversion occur throughout the life of cows [95,96]. A recent study investigated whether CLA supplementation alters muscle metabolism or mobilization of muscle protein [60]. The authors showed that CLA supplementation from -63 d antepartum to 63 d postpartum did not alter the weight and area of longissimus muscle nor the number, size, and type of muscle fibers. The low content of t10,c12 CLA in longissimus muscle may lead to the results or the muscle has already recovered at time of slaughter. The content of t10,c12 CLA in skeletal muscle is different in individuals. Von Soosten et al. [43] showed that t10,c12 CLA could not be detected in longissimus muscle in the early lactating cows. The study of Yang et al. [97] investigated the effect of dietary CLA supplemented during early lactation on the regulation of specific signaling components of protein turnover in the skeletal muscle of dairy cows and found activation of catabolic and anabolic signaling pathways, suggesting increased protein turnover. Muscle protein mobilization in lactating cows is not generally observed during lactation and not much studied. Thus, the knowledge about CLA effects in muscle tissue is limited and further research is needed, but it may be more important in beef cattle.

## 4. Effects of CLA Supplementation in Beef Cattle

Beside milk and milk products, meat is a major source of CLA for humans [98]. Cattle individuals can produce distinct amounts of endogenous CLA, and the capacity of CLA synthesis is genetically determined [99]. However, gender, season, breed and feeding, in particular supplements, all affect the CLA content in cattle tissues [100,101]. Studies in growing ruminants have examined the potential of forage species, forage conservation method and dietary plant oils, oilseeds, fish oil and marine algae supplements to alter muscle fatty acid composition (reviewed by Shingfield [102]). It was shown that the relative increase in t10,c12 CLA in the beef was higher than that of c9,t11 CLA upon supplementation [103,104]. Thus, it is important to consider effects of the increased amounts of this isoform in respective tissues and consequences for tissue accretion, growth and meat quality. Studies in species, such as pigs, mice, and rats, indicated that CLA supplementation reduced the content of subcutaneous fat and increased IMF content [105,106,107]. In rodents, CLA mixture increased lean meat weight and reduced the fat tissue weight [108,109]. However, the effects of t10,c12 CLA or CLA mixture on lipid metabolism and changes in tissues during the growing or finishing of beef cattle are not clear. Only few studies were reported, as summarized in Table 3, and most of them found no or inconsistent effects of supplemented CLA on growth, carcass composition or meat quality. Schlegel et al. [110] showed that 1.9 g and 4.8 g CLA supplementation significantly increased t10,c12 CLA percentage 1.63-fold or 2-fold in longissimus muscle, but 2-fold or 4-fold in the subcutaneous fat of Simmental heifers. Thus, lipid metabolism in muscle and adipose tissue could both be influenced by supplemented CLA. However, studies indicated that the supplemented CLA had minor effects on the growth of young beef cattle. Holstein bulls, which were supplemented with 2% CLA for ~123 days and a body weight of 458 kg, had a slightly increased growth rate and similar meat quality as control bulls [111,112,113]. Moreover, 2% CLA supplementation in crossbred heifers of Angus and Hereford did not change carcass traits, SCF deposition, and IMF content, but improved growth and slightly impaired meat quality compared to control animals [114,115,116]. However, in studies with Charolais × Limousin heifers [117] and Piemontese bulls [118,119], the growth performance and meat quality were similar to the control bulls, even though the t10,c12 CLA increased 20-fold in the muscle tissue of Piemontese bulls. Abomasal infusion of CLA in Angus steers elevated plasma levels of both isoforms, but only t10,c12 CLA was increased in SCF in the study of Choi et al. [120]. In concordance with earlier studies [114,121,122], this may indicate that the amount of this isoform, which has higher biological activity for nutrient partitioning, can more easily be manipulated in adipose and muscle tissue than the amount of c9,t11 CLA.

When CLA is supplemented in beef cattle during the fattening period, t10,c12 CLA inhibited fat accumulation in the subcutaneous fat tissue and reduced the back fat thickness [12]. Moreover, surplus energy may be used in muscle to increase de novo fatty acid synthesis and enhance IMF storage. This would improve marbling and meat quality, while simultaneously reducing subcutaneous fat. Concordantly, Zhang et al. [12] reported that the back fat thickness, subcutaneous fat and visceral fat percentage all decreased, whereas the IMF content significantly increased in a study with crossbreeds of Chinese Yellow cattle and Simmental cattle at 16 months of age, supplemented with 2% CLA for 60 days. Furthermore, the expression of genes related to lipogenesis was upregulated in the muscle, supporting IMF deposition, while lipolysis enzymes, such as LPL, HSL, and CPT1, were upregulated in subcutaneous adipose tissue [12]. However, no further studies could be found confirming this effect of CLA supplementation in beef cattle and further research is necessary to verify it. In contrast to dairy cows, the beneficial effect of CLA supplementation in beef cattle is limited and not recommendable in view of the higher expense for such a supplementation.

## 5. Effects of CLA Supplementation in Adipogenic and Myogenic Cells

Supplemented CLA influences cellular processes in adipose and muscle tissues, such as dedifferentiation and regeneration in adult cattle, as well as proliferation and differentiation in growing animals and in the developing fetus, when pregnant cows are supplemented. As shown in the studies of Uken et al. [73] and Dahl et al. [123], maternally supplemented CLA or essential fatty acids will be transferred to the fetus and can change the blood and tissue concentrations of CLA. Thus, CLA may influence the growth and development of cells in different tissues of the fetus.

Cell culture models can help to improve our understanding of cellular processes behind the observed effects of CLA in adipose and muscle tissue. Primary cells, isolated from different adipose and muscle tissues of various species, and established cell lines, such as 3T3L1 and C2C12 or L6 myoblasts were used to investigate CLA effects on proliferation and differentiation as well as on lipid metabolism and associated gene expression.

### 5.1. CLA Effects in Adipogenic Cells

Effects of CLA supplementation on the viability and proliferation of primary cells or cell lines are not consistent over studies. Viability of 3T3-L1 cells was inhibited by different concentrations of CLA mixtures, consisting of 41% c9,t11/t9,c11, 44% t10,c12, and 10% c10,c12 isomers, by 8%, 12%, 31%, and 36%, for 0.5, 1, 5, or 10 mg/L respectively, in the study of Satory et al. [124]. Another study showed that 0–200 µM CLA mixtures, mainly c9,t11 and t10,c12 CLA, inhibited 3T3L1 cell proliferation in a dose-dependent manner, whereas linoleic acid had no effect on viability [125]. McNeel and Mersmann [126] reported that 50 µM CLA mixtures or isomers did not reduce the number of cells in a cell culture model with porcine stromal-vascular cells (SVCs), derived from subcutaneous fat, while proliferation of porcine SVCs was inhibited by 50 µM CLA mixtures or t10,c12 CLA in the study of Zhou et al. [127]. Brandebourg et al. [128] found that only t10,c12 CLA (0–25 µM) reduced the number of porcine SVCs in a dose-dependent manner, whereas CLA mixtures or c9,t11 CLA had no effect on cell number at concentrations of 0–100 µM. In contrast, Jiang et al. [105] showed that 50–350 µM CLA isomers promoted the proliferation of porcine SVCs, while a concentration of 400 µM of CLA isomers caused inhibition.

The effects of CLA mixtures or t10,c12 CLA on adipocyte differentiation are similarly inconsistent. It was reported that differentiation of 3T3-L1 adipocytes as well as porcine and bovine adipocytes was inhibited by supplementation with t10,c12 CLA via the downregulation of adipogenic and lipogenic genes, such as *FASN*, *PPARG*, *CEBPA*, *LPL*, *SREBP1C* and *SCD* [125,128,129,130]. The *SCD* gene plays a key role in adipocyte differentiation, fat deposition, and MUFA content [131] and multiple studies showed that CLA reduced *SCD* expression or activity in ruminants [54,132,133,134]. In contrast, Satory et al. [124] reported that CLA promoted differentiation of 3T3-L1 adipocytes, while McNeel and Mersmann [126] showed that 50 µM t10,c12 CLA increased lipid droplet content in porcine SVCs at two days of differentiation, but did not affect overall adipocyte differentiation or the expression of *PPARG* and *LPL* over the entire differentiation period.

Studies in the 3T3L1 cell line proved different effects of individual CLA isoforms. Evans et al. [135] demonstrated a triglyceride-lowering effect of t10c12 CLA in contrast to greater TG accumulation after c9,t11 CLA treatment. Studies of Park et al. [16] indicated reduced LPL activity and increased lipolysis in 3T3-L1 cells after t10,c12 CLA treatment, whereas Evans et al. [125] reported induced apoptosis. Subsequent studies identified reduced adipogenesis related gene expression and increased fatty acid oxidation as a possible cause for the triglyceride-lowering effect of t10,c12 CLA [129,136], whereas Yeganeh et al. [137] concluded that the decrease in lipid droplets caused by t10,c12 CLA is the result of an inhibition of lipid droplet production during adipogenesis rather than a stimulation of lipolysis. In the study of Zhou et al. [127] with SVCs from subcutaneous and muscle tissue of neonatal pigs, the results indicated that t10c12 CLA, but not c9,t11 CLA, inhibited adipogenesis in subcutaneous cells, while both isomers promoted intramuscular adipogenesis. In bovine preadipocytes, t10,c12 CLA reduced the differentiation by inhibition of lipogenesis and *SCD1* expression [133,138]. Evans et al. [136] reported that 50 µM t10,c12 CLA enhanced de novo lipogenesis from glucose and oleic acid. The same study further demonstrated that t10,c12 CLA slightly increased triglyceride synthesis but strongly stimulated basal fatty acid oxidation without affecting lipolysis, collectively leading to reduced fat accumulation in adipocytes. Several other studies indicated that t10,c12 CLA did not enhance lipolysis or the expression of related genes such as *ATGL* and *HSL*, but rather suppressed de novo fatty acid synthesis, thereby inhibiting cellular fat deposition [60,137,139].

It can be concluded that low concentrations of t10,c12 CLA, or treatment during early differentiation, may not suppress fat accumulation because t10,c12 CLA is incorporated as a normal fatty acid and esterified into triglycerides, thereby potentially promoting differentiation. This is supported by Lengi and Corl [138], who showed that exogenous inducers containing medium- and long-chain fatty acids in serum-free medium effectively promoted bovine preadipocyte differentiation compared to conventional inducers, indicating that fatty acid supplementation can support adipocyte differentiation even in the absence of acetate or glucose. Several studies have reported that linoleic acid, c9,t11 CLA, and t10,c12 CLA all promoted adipocyte differentiation [124,139,140]. These fatty acids may be incorporated into triglycerides without exerting inhibitory effects on fat accumulation. Evans et al. [135] provided data on the distribution of t10,c12 CLA in phospholipid and neutral lipid fractions of 3T3-L1 cells. When supplemented at 10 µM, t10,c12 CLA was primarily stored in the neutral lipid fraction, whereas at 50 µM, it was distributed in both phospholipid and neutral lipid fractions [135]. Fatty acids in the phospholipid fraction are more likely to exert regulatory functions than those in neutral lipids. These findings suggest that at low concentrations, t10,c12 CLA potentially facilitates fat deposition when it is preferentially incorporated into neutral lipids during early differentiation. In contrast, at higher concentrations, it is also incorporated into phospholipids, where it may exert inhibitory effects on adipocyte fat deposition. Thus, both the dose and the intracellular localization of t10,c12 CLA are critical factors influencing resulting effects. Despite the use of different cells and cell lines in these studies, a strong dependence of the observed effects on the applied concentrations of t10,c12 CLA or on the composition of CLA mixes was observed. However, the underlying mechanisms, such as regulation of lipid accumulation, lipogenesis and lipolysis, remain incompletely elucidated due to the heterogeneity of the study designs.

### 5.2. CLA Effects in Myogenic Cells

The observed anti-obesogenic effects of t10,c12 CLA and the influence on insulin sensitivity, glucose uptake and intramuscular fat deposition, led to several studies aiming at elucidation of CLA effects, either mixtures or individual isomers, on myocytes. Most of this research has been conducted using C2C12 and L6 myoblast cell lines, both of which undergo proliferation and differentiation into myotubes under in vitro conditions. Contrasting results were also reported with those cells and further research is recommended.

Lee et al. [141] demonstrated that proliferation and differentiation of C2C12 cells were stimulated by c9,t11 CLA and inhibited by t10,c12 CLA. In contrast, the studies by Kim and Park [142] showed that t10,c12 CLA improved the proliferation and differentiation of the C2C12 cell line, increased the mitochondrial biogenesis by promoting the *PGC1A* gene and protein expression and AMPK pathway in a dose-dependent manner. Mohammadi et al. [143] reported that 50–150 µM CLA mixtures promoted C2C12 cell proliferation. Similarly, Hurley et al. [144] observed that 0–200 µM t10,c12 CLA stimulated L6 myoblast proliferation, while inhibiting differentiation in a dose-dependent manner. Ovine satellite cells that were stimulated with CLA increased proliferation, but differentiation remained unchanged [145]. Moreover, Larsen et al. [146] found that 25 µM CLA mixtures or t10,c12 CLA suppressed the differentiation of human muscle cells and downregulated the expression of *MYOG*, *MYOD*, creatine kinase, and *MYHCIIx*. Using 50 µM t10,c12 CLA in C2C12 cells, Hommelberg et al. [147] also demonstrated inhibition of myogenic differentiation and reduced expression of creatine kinase, *Myog*, *MyhcIIb*, and *Glut4.* In contrast, Mohankumar et al. [148] showed that 60 µM t10,c12 CLA induced Ca^2+^ release and promoted phosphorylation of CaMKII, AMPK, and AS160, leading to GLUT4 translocation to the plasma membrane and enhanced glucose uptake in L6 myoblasts. This treatment also increased cellular ROS activity without affecting SAPK/JNK phosphorylation [148]. In another study, the same group reported that 60 µM t10,c12 CLA increased phosphorylation of AMPK-α at Thr172 and ACC at Ser79, further supporting its role in glucose uptake regulation [149]. Kim and Park [142] found that CLA mixtures induced AMPK-α phosphorylation and elevated expression of *Pgc1a*, *Nrf1*, and *Tfam*, promoting mitochondrial biogenesis in C2C12 cells. Qin et al. [150] showed that c9,t11 CLA phosphorylated AMPK, enhanced glucose oxidation, and attenuated palmitic acid-induced insulin resistance in C2C12 cells. As AMPK acts as a key cellular energy sensor and regulates glucose and lipid metabolism in response to tissue energy demands [151], these studies collectively suggest that CLA mixtures and individual isomers modulate muscle cell metabolism partly through activation of the AMPK pathway. However, all available studies targeted immortalized cell lines derived from rodents while comprehensive studies investigating CLA effects on bovine muscle cells are still lacking. Consequently, it remains unclear to which extent the reviewed results are transferable to bovine species. Further research is necessary to better understand CLA supplementation induced cellular and metabolic processes in skeletal muscle of transition cows and growing cattle.

In summary, results from experiments with myogenic or adipogenic cells do not comprehensively elucidate the cellular processes in response to CLA. Further research, especially on primary cells from bovines, are required to facilitate a better understanding of the supplementation effects.

## 6. Conclusions

Supplementation with CLA, in particular with t10,c12 CLA, can improve the energy balance of high yielding dairy cows through milk fat depression. It seems that it is more efficient in cows during the transition period than in later lactation periods, although a lag phase of a few days can be observed after parturition before CLA effects become apparent. T10,c12 CLA reduces the content and yield of milk fat, especially fatty acids with less than 16 carbon atoms, and decreases milk energy output and body mass mobilization. The saved energy, mediated by t10,c12 CLA, can be repartitioned in three directions, namely alleviation of the negative energy balance, increase in milk yield, or accretion of muscle or adipose tissue. The energy repartitioning directions may be determined by genetics and metabolic status. In non-lactating and growing or finishing cattle, CLA supplementation may affect the body composition, reducing, e.g., subcutaneous fat. However, further research is necessary to fully understand the cellular processes behind observed changes. Furthermore, for practical application, more research is needed to understand the long-term health effects in supplemented cattle, to find the optimal doses and time of supplementation and to consider economical aspects of CLA supplementation.

## Figures and Tables

**Table 2:** Effects of t10,c12 CLA supplementation on plasma profiles, BFT, and liver triglycerides.

Cows	Supplementation, g/d	Method	Period	NEFA	BHBA	TG Blood	TC	Glucose	Insulin	BFT	TG Liver	RQUICKI	Reference
m-Holstein	10.2	infusion	−63 d to 63 d	↓	↓	↓	↔	↑	↑	↑	↓	/	[56,69]
m-Holstein	4.7/9.9/14.8	dietary	−10 d to 21 d	↔	/	/	/	↔	/	/	/	/	[5]
m-Holstein	7.5	dietary	−20 d to 21 d	↓	↓	/	/	↑	↑	/	/	/	[45]
m-Holstein	6.8	dietary	−21 d to 28 d	↔	↑	˄	↑	↔	˄	/	/	˅	[62]
m-Holstein	12	dietary	−21 to 60 d	↔	↓	↑	↑	↑	↑	/	/	/	[61]
m-Holstein	6.8	dietary	−21 d to 91 d	↓	↓	↓	↔	↔	/	/	↓	/	[38]
pm-Holstein	3.8	dietary	−21 d to 98 d	↓	↔	↔	↔	/	/	/	↔	/	[63]
m-Holstein	7.6	dietary	1 d to 70 d	↓	/	/	/	↑	↑	/	/	/	[74]
p-Holstein	6	dietary	1 d to 105 d	↔	↔	/	/	↔	/	↓	/	/	[44]
m-Holstein	4.6/9.2/18.4	infusion	126 d for 6 w	↔	↔	↔	↓	↔	↑	↔	/	/	[75]
m-Holstein	10	infusion	non-lact. for 3 d	↑	↑	↔	↔	↔	↑	/	/	/	[76]

NEFA: non-esterified fatty acids; BHBA: β-hydroxybutyric acid; TG: triglyceride; TC: total cholesterol; BFT: back fat thickness; RQUICKI: revised quantitative insulin sensitivity check index; m: multiparous; p: primiparous; /: no data; ↓ decrease; ˅ decrease trend; ↑ increase; ˄ increase trend; ↔ no change.

**Table 3:** Effects of CLA supplementation on tissue concentration of CLA isomers, growth, carcass traits, amounts of SCF and IMF, as well meat quality in beef cattle.

Breed	Gender	Age	Supplementation	Method	Duration	t10/c12 CLA Conc.		c9/t11 CLA Conc.		Growth	Carcass traits	SCF	Meat Quality	IMF	Reference
						LM	SCF	LM	SCF						
Simmental	heifers	5 mo	0/100/250 g/d	dietary	16 wk	↑	↑	↔	↔	↔	↔	↔	↔	↔	[110]
Holstein	bulls	52 d	5/10 g/kg DM	dietary	63 d	↑	/	↔	/	↔	/	/	/	↔	[122]
Angus cross	steers	weaning + 195 d	84 g/d	dietary	130 d	↑	↑	↔	↔	/	/	/	↔	/	[121]
Holstein	bulls	199 d	2%	dietary	123 d	↔	↑	↑	↔	↑	↔	↔	↔	↔	[111,112,113]
Angus × Hereford	heifers	13 mo	2%	dietary	32/60 d	↑	↑	↔	↑	↑	↔	↔	↓	↔	[114,115,116]
Charolais × Limousin	heifers	11 mo	25/50 g/d	dietary	180 d	/	/	/	/	↔	↔	↔	↔	/	[117]
Piemontese	bulls	202 d	80 g/d	dietary	332 d	↑	↔	↑	↔	↔	↔	↓	↔	↔	[118,119]
Beef on dairy	bulls, heifers	196/191 d	8/80 g/d	dietary	324/269 d	/	/	/	/	↑	↑	↔	↔	↔	[100]
Yellow Breed × Simmental	/	16 mo	2%	dietary	60 d	/	/	/	/	↔	↔	↓	/	↑	[12]
Angus	steers	12–16 mo	100 g/d	infusion	14 d	/	↑	/	↔	↔	/	/	/	/	[120]

Conc.: concentration; LM: longissimus muscle; SCF: subcutaneous fat; IMF: intramuscular fat; /: no data; ↓ decrease; ↑ increase; ↔ no change.

## Data Availability

No new data were created or analyzed in this study. Data sharing is not applicable to this article.

## Abbreviations

The following abbreviations are used in this manuscript:

Abbreviation		Abbreviation	
AA	Amino Acid	IGFBP	Insulin Like Growth Factor Binding Protein
*ACACA*	Acetyl-CoA Carboxylase Alpha	IMF	Intramuscular Fat
ACC	Acetyl-CoA Carboxylase	LA	Linoleic Acid
ACLY	ATP Citrate Lyase	LCFA	Long Chain Fatty Acid
ALA	α-linolenic acid	LPL	Lipoprotein Lipase
AMPK	AMP-Activated Protein Kinase	MEC	Mammary Epithelial Cell
APO	Apolipoprotein	MFD	Milk Fat Depression
AS160	Akt Substrate Of 160 KDa	MFG	Milk Fat Globule
ATGL	Adipose Triglyceride Lipase	mTOR	Mechanistic Target Of Rapamycin Kinase
BFT	Back Fat Thickness	MUFA	Monounsaturated Fatty Acids
BHBA	β-Hydroxybutyric Acid	*MYHC*	Myosin Heavy Chain
BW	Body Weight	*MYOD*	Myogenic Differentiation
c9,t11 CLA	cis-9/trans-11 CLA	*MYOG*	Myogenin
CaMK	Calcium/Calmodulin Dependent Protein Kinase	NEFA	Non-Esterified Fatty Acid
*CD36*	Fatty Acid Translocase	*PLIN*	Perilipin
*CEBPA*	CCAAT Enhancer Binding Protein Alpha	*PPARG*	Peroxisome Proliferator Activated Receptor Gamma
CLA	Conjugated Linoleic Acid	PUFA	Polyunsaturated Fatty Acid
CPT	Carnitine Palmitoyltransferase	RQUICKI	Revised Quantitative Insulin Sensitivity Check Index
DEG	Differentially Expressed Gene	SAPK/JNK	Mitogen-Activated Protein Kinase
DMI	Dry Matter Intake	*SCD*	Stearoyl-CoA Desaturase
EB	Energy Balance	Ser	Serin
ECM	Energy Corrected Milk	*SREBP1*	Sterol Regulatory Element-Binding Protein 1
FA	Fatty Acid	SVC	Stromal-Vascular Cells
FABP4	Fatty Acid Binding Protein 4	t10,c12 CLA	trans-10/cis-12 CLA
FAS/FASN	Fatty Acid Synthase	TC	Total Cholesterol
Glut4	Glucose Transporter 4	TG	Triglyceride
HDL	High-Density Lipoprotein	Thr	Threonin
